# Tobacco product cessation and prenatal care utilization: A Pregnancy Risk Assessment Monitoring System phase 8 study of conventional cigarette, e-cigarette, and dual-use associated behaviors

**DOI:** 10.1371/journal.pone.0343423

**Published:** 2026-03-03

**Authors:** Rathika Damodara Shenoy, Michael Weitzman, Juan M. Acuña

**Affiliations:** 1 Department of Clinical Medicine, American University of Antigua College of Medicine, Coolidge, Antigua & Barbuda; 2 Department of Pediatrics, Kasturba Medical College Mangalore, Manipal Academy of Higher Education, Manipal, Karnataka, India; 3 Department of Pediatrics, New York University Grossman School of Medicine, New York, United States of America; 4 Department of Global Health, American University of Antigua College of Medicine, Coolidge, Antigua & Barbuda; 5 Department of Environmental Health, UAlbany College of Integrated Health Sciences, Albany, New York, United States of America; 6 Department of Global Health, American University of Antigua College of Medicine, Coolidge, Antigua & Barbuda; 7 Department of Epidemiology, Robert Stempel College of Public Health & Social Work, Florida International University, United States of America; University of Health Sciences (Istanbul, Türkiye), TÜRKIYE

## Abstract

**Background:**

Pregnancy is a critical window for tobacco cessation; socio-demographic correlates and prenatal care (PNC) utilization behaviors associated with quitting e-cigarette and dual use with conventional cigarettes remain understudied.

**Objective:**

To examine the influence of socio-demographic characteristics and PNC utilization in the cessation of cigarette, e-cigarette, and dual-use during pregnancy, using Pregnancy Risk Assessment Monitoring System (2016–2022) data.

**Methods:**

We analyzed data from 223,793 respondents (weighted count = 11,475,844) with singleton births who reported cigarette and/or e-cigarette use during the three months before and the last three months of pregnancy. Socio-demographic and PNC cessation correlates for cigarette, e-cigarette, and dual-use were examined versus continuation using logistic regression analysis. The Adequacy of Prenatal Care Utilization (APNCU) Index assessed PNC. Associations were expressed as adjusted odds ratios with 95% confidence intervals [AOR (95%CIs)].

**Results:**

Tobacco product use declined from 16.9% pre-pregnancy to 7.5% by late pregnancy. Pregnancy-associated cessation rates were 53.7% for cigarettes, 81.0% for e-cigarettes, and 48.1% for dual-use. Primiparity was associated with higher odds of quitting across all groups: cigarettes [1.8 (1.6‒2.0)], e-cigarettes [1.6 (1.2‒2.1)], and dual-use [2.3 (95% CI: 1.7‒3.1)]. Black race and Hispanic ethnicity were positively associated with cessation of cigarettes and dual-use, while Black race was also associated with higher odds of EC cessation. A higher smoking frequency was associated with reduced cessation odds of cigarette [0.2 (0.2‒0.3)] and dual-use [0.3 (0.2‒0.4)], while a higher vaping frequency was associated with reduced e-cigarette cessation [0.3 (0.2‒0.5)]. Inadequate APNCU Index was associated with lower odds of quitting cigarettes [0.6 (0.5‒0.7)] and dual-use [0.6 (0.4‒0.9)], but showed no significant association with e-cigarette cessation [0.9 (0.6‒1.5)].

**Conclusions:**

Distinct socio-demographic and PNC factors influence cessation patterns by product type. Findings underscore potential opportunities to integrate PNC with targeted cessation support, particularly for high-risk groups amid rising e-cigarette and dual use during pregnancy.

## Introduction

Conventional cigarette (CC) smoking during pregnancy is linked to adverse maternal and fetal outcomes, such as pregnancy loss, reduced fetal growth, and long-term neurodevelopmental consequences for the child. Smoking cessation during pregnancy significantly reduces the risks of fetal growth restriction and small-for-gestational-age births [1,2]. In the U.S., the prevalence of smoking during pregnancy has been declining over time across all socio-demographic groups from 17.4% in 2015 to current estimates ranging from 5.4% to 8.4% [3–8].

Although exclusive CC use remains the most common form of tobacco use among women of childbearing age, use of non-combustible tobacco extracts containing nicotine, particularly electronic cigarettes (EC), is on the rise [9]. ECs are classified as alternative tobacco products, even though newer generations of electronic nicotine delivery systems (ENDS) are compatible with vaping substances such as synthetic nicotine and cannabis [10,11]. Popular EC brands among young adults often contain nicotine salts, which allow higher nicotine concentrations due to their increased solubility in e-liquid [11].

The prevalence of EC use among pregnant women has increased from 1.9% in 2016 to 3.8% in 2018, with 0.6% reporting daily use [12]. Data from the U.S. Pregnancy Risk Assessment Monitoring System (PRAMS) show that the prevalence of exclusive EC use among pregnant adolescents increased from 0.8% in 2016 to 4.1% in 2021 [13]. The use of ENDS during pregnancy is more common than hookah, cigar, or smokeless tobacco products [14]. Pregnant women perceive ECs as a safer alternative to traditional cigarettes, despite limited evidence supporting their safety during pregnancy [1,15–18].

Dual use of CC and EC is the second most prevalent pattern of nicotine use after exclusive cigarette smoking among women of childbearing age [19]. About 46.3% of EC users and 13.9% of CC users report dual use of EC and CC [12]. The overall prevalence of dual use during pregnancy is approximately 1.1%. Data from PRAMS indicate a fluctuating dual-use prevalence of 0.6%−1.6% among pregnant adolescents during 2016–2021 [20]. With rising trends in EC and dual use, the American College of Obstetricians and Gynecologists cautions that women may not recognize ECs as a form of tobacco or nicotine use, contributing to continued exposure during pregnancy [2]. Emerging data from the PRAMS suggest adverse pregnancy outcomes associated with EC and dual-use of EC and CC [13,21–26].

Pregnancy itself promotes tobacco cessation among women. Longitudinal data (2013–2017) from the Population Assessment of Tobacco and Health (PATH) Study show that 76.2% of EC users, 45.5% of CC users, and 43.1% of dual users quit during pregnancy [27]. Early and adequate prenatal care (PNC) visits provide opportunities for education and counseling on health-enhancing behaviors. A meta-analysis of global studies indicated that adequate PNC was associated with higher odds for smoking cessation [28]. Standard PNC during the first trimester contributed to a 3.4% decline in smoking trends [8]. Less than 10% of women require a formal cessation program [8,29,30].

Many of the correlates of women who quit smoking during pregnancy have been well documented [8,14,27,31–33]. While much has been done on smoking cessation, there remains a significant gap in understanding the cessation patterns among EC or dual users, particularly how socio-demographic factors and PNC utilization influence their cessation behavior. We hypothesize that the demographic, behavioral, and health-seeking correlates of women who quit EC or dual use during pregnancy differ from those who continue their use. The study investigated how socio-demographic characteristics and PNC utilization behaviors were associated with the cessation of CC, EC, and dual use during pregnancy, using data from PRAMS Phase 8 (2016–2022).

## Methods

This study analyzed PRAMS Phase 8 data from the Centers for Disease Control and Prevention (CDC) Surveillance System. PRAMS data were collected through complex, stratified, state-based sampling between 2016 and 2022. Each participating state probabilistically sampled between 1,000 and 3,000 women from birth certificate records each year, ensuring a representative state sample. State-specific questionnaires were mailed 2‒4 months after delivery, with follow-up calls for non-responders to complete interviewer-administered versions [34]. PRAMS provided de-identified birth certificate-linked variables for analysis along with self-administered or interviewer-administered data.

### Variables

**Fig 1:**
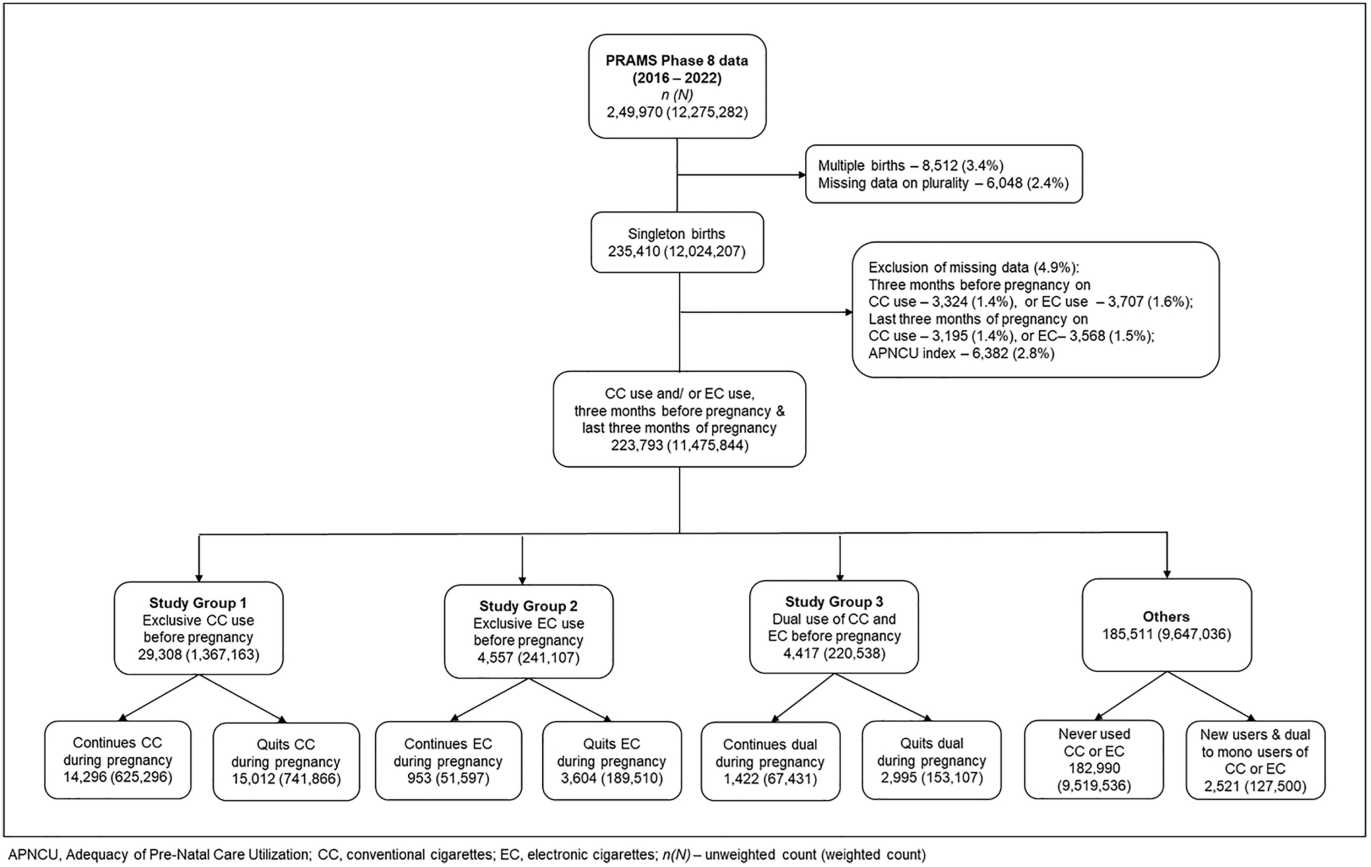
Flow chart of study population selection.

We included 223,793 respondents with singleton births (corresponding to a weighted count of 11,475,844) who provided information on CC use (core questions 20 and 21) and EC use (core questions 24 and 25) during the three months before and the last three months of pregnancy (Fig 1). Exclusion criteria for the analyses included multiple births and missing data on plurality, CC use, EC use, or the Adequacy of PNC Utilization (APNCU) Index.

Multiple births and cases with missing information on plurality (~14,000) were excluded because multiple gestations differ in obstetric risk classification and management, and their inclusion would introduce structural heterogeneity. In addition, singleton pregnancies with missing APNCU information were excluded because APNCU was the primary exposure of interest in this study (~6,000). Complete information on CC and EC use in the three months before pregnancy and the last three months of pregnancy was also required to assess pregnancy-associated tobacco behaviors, and observations lacking these data were excluded (~5000).

The data were recoded into three mutually exclusive study groups: those who quit or continued using CC, those who quit or continued using EC, and those who quit or continued dual use of CC and EC during pregnancy. Pregnancy-associated cessation was defined as self-reported discontinuation of CC and/or EC use during the last three months of pregnancy relative to its use in the three months before pregnancy, consistent with PRAMS core questionnaire design.

**Fig 2:**
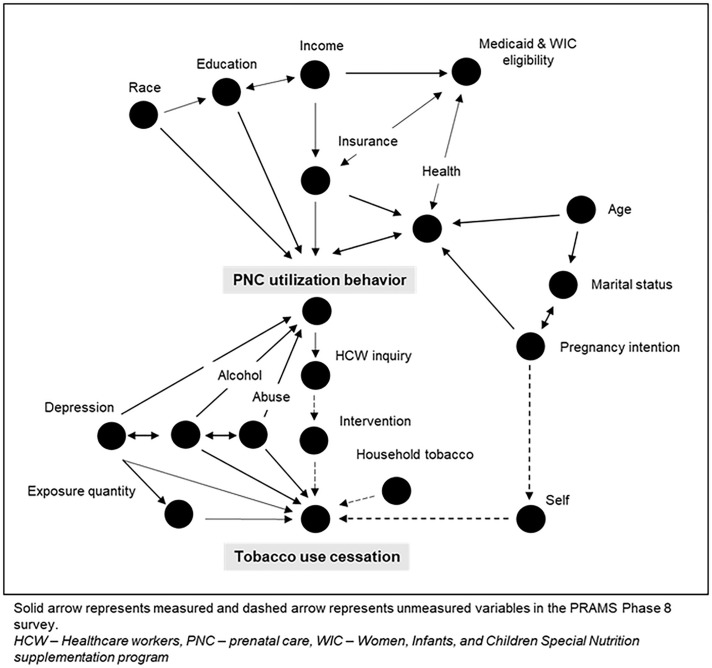
Directed acyclic graph showing the relationships between sociodemographic, behavioral, health-seeking, and nicotine cessation behaviors during pregnancy.

The study's primary outcome was to examine the socio-demographic and PNC utilization correlates associated with the cessation of CC, EC, and their dual use during pregnancy. A directed acyclic graph was used to illustrate the relationships between maternal cessation behavior and socio-demographic, behavioral, and health-seeking correlates (Fig 2). The APNCU Index assessed PNC utilization adequacy.

### Bias

PRAMS provides trimester-specific data on CC use across all three trimesters based on birth certificate information; however, EC use is assessed by recall only for the first and third trimesters. This can potentially misclassify individuals who quit CC in the first or second trimester but relapse in the third trimester as ‘continuers,’ and does not capture ‘reducers’ who significantly cut down, but did not completely quit. Additionally, given that smoking or ENDS use data are self-reported, there is a risk of nonresponse and recall bias. The APNCU, also known as the Kotelchuck Index, primarily evaluates the timing of initiation and the frequency of PNC visits [35]. It does not assess the quality of care or whether health screening, health education, or appropriate interventions for cessation of CC and EC use were offered. It is plausible that even with adequate PNC visits as assessed by APNCU, the healthcare workers (HCW) may not be routinely screening for or effectively counseling on EC use, as the focus is traditionally on combustible tobacco products. It is also important to note that PRAMS surveys include only women with live births, potentially underrepresenting high-risk pregnancies or pregnancy loss due to tobacco use during pregnancy, leading to survivorship bias.

### Statistical methods

We used the IBM Statistical Package for Social Studies (SPSS) version 25 with the complex sampling and testing add-on (survey package). PRAMS data is statistically weighted to account for different sampling rates across strata and nonresponse.^34^ Socio-demographic variables, including age, education, ethnicity, and race, were reclassified into aggregated categories as presented in S1 Table in S2 File to simplify analysis, avoid overfitting, and improve statistical power. Ethnicity and race data were classified as Hispanic, non-Hispanic White, Black, and Other (including American Indian, Alaskan Native, Pacific Islander, Asian, or multi-ethnic).

Numerical data on previous pregnancies were classified into primiparous and multiparous. Responses about pregnancy intention were categorized as intended and unintended. Numerical data on the initiation of PNC and the number of visits were also analyzed for their independent associations with tobacco product cessation. Cigarette exposure was recoded into pack equivalents daily, while EC use was categorized by weekly use (S1 Table in S2 File). Alcohol use three months before pregnancy was reorganized as none or occasional (fewer than four drinks per week), moderate (4‒7 drinks per week), and heavy (more than seven drinks per week).

Cessation and continuation of CC, EC, and dual-use during pregnancy were analyzed separately for each tobacco product group. Period proportion was calculated as the weighted proportion of respondents who reported continued or discontinued use during Phase 8. The pregnancy-associated cessation rate for each tobacco product was calculated as the proportion of individuals who reported quitting by the third trimester among those who reported product use during the three months before pregnancy.

Socio-demographic and PNC cessation correlates were examined versus continuation using unadjusted and adjusted approaches. Proportions and odds ratios (OR) were calculated for each covariate using weighted cross-tabulations and simple logistic regression to assess their independent association with cessation behavior. Multiple logistic regression assessed the combined effect of multiple covariates on cessation behavior, with adjusted odds ratios (AORs) calculated. A 95% confidence interval (CI) was provided for all estimates, and a *p*-value <0.05 was considered significant.

Confounding was adjusted for, and interactions were tested using multiple logistic regression to examine the combined effect of two or more predictor variables on the outcome. Missing socio-demographic data were excluded from the statistical analysis. For sensitivity analyses, we compared the characteristics of the included and excluded populations and also evaluated the stability of the logistic regression model performance across survey-year subpopulations.

### Ethical statement

The study involves a secondary analysis of de-identified PRAMS Phase 8 (2016−22) data, accessed on November 13, 2024, through the CDC portal (http://pramsarf.cdc.gov). The Institutional Review Board reviewed the study protocol and determined that the study qualified for exemption under applicable regulatory criteria, per the ethical principles outlined in the 1964 Declaration of Helsinki and its later amendments. This report adhered to the STROBE guidelines for cross-sectional studies.

## Results

The period prevalence of exclusive and dual use of CC and EC declined from 16.9% before pregnancy to 7.5% in late pregnancy (S2 Table in S2 File). Among women who exclusively smoked before pregnancy (12.1%), 6.5% reported quitting by the third trimester, corresponding to a pregnancy-associated cessation rate of 53.7%. For EC users, 2.1% reported use before pregnancy, and 1.7% discontinued use during pregnancy, with a higher cessation rate of 81.0%. Among dual users (2.7%), 1.3% quit both products during pregnancy, resulting in a cessation rate of 48.1%. Higher cessation rates were observed among women who smoked half a pack or less of cigarettes and among those who reported occasional EC use (Table 1). Cessation of CC use was relatively consistent across trimesters; however, a greater proportion of women who smoked 11−20 cigarettes per day quit during the first trimester, whereas those who smoked 1−10 cigarettes per day were more likely to quit only during the last three months of pregnancy. In addition, 4.3% (95%CI: 4.1–4.4) of CC users reduced the number of cigarettes smoked per day by the third trimester, without achieving complete cessation.

**Table 1:** Cigarettes and/or e-cigarette exposure before and during pregnancy.

Frequency	Weighted proportion % [95%CI]				Proportion quitting % [95%CI]
	Three months before pregnancy	First trimester	Second trimester	The last three months of pregnancy	
Cigarettes per day					
None	85.2 [84.9,85.4]	93.7 [93.6,93.9]	94.7 [94.5,94.8]	93.1 [93.0,93.3]	
1-10	10.1 [9.9,10.3]	4.6 [4.5,4.8]	4.3 [4.2,4.5]	5.8 [5.6,5.9]	65.9 [64.9,66.8]
11-20	3.6 [3.5,3.7]	1.5 [1.4,1.6]	0.9 [0.8,1.0]	0.9 [0.8,0.9]	30.7 [29.1,32.3]
>20	1.1 [1.1,1.2]	0.2 [0.2,0.2]	0.1 [0.1,0.1]	0.2 [0.2,0.2]	26.0 [23.4,28.9]
E-cigarettes per week					
None	95.1 [95.0,95.3]			98.5 [98.4,98.6]	
Occasional	1.6 [1.5,1.6]			0.5 [0.4,0.5]	79.2 [77.0,81.3]
Some days	0.6 [0.5,0.6]			0.2 [0.2,0.2]	76.1 [72.4,79.4]
Every day	2.7 [2.6,2.8]			0.8 [0.8,0.9]	71.2 [69.4.73.0]

**Fig 3:**
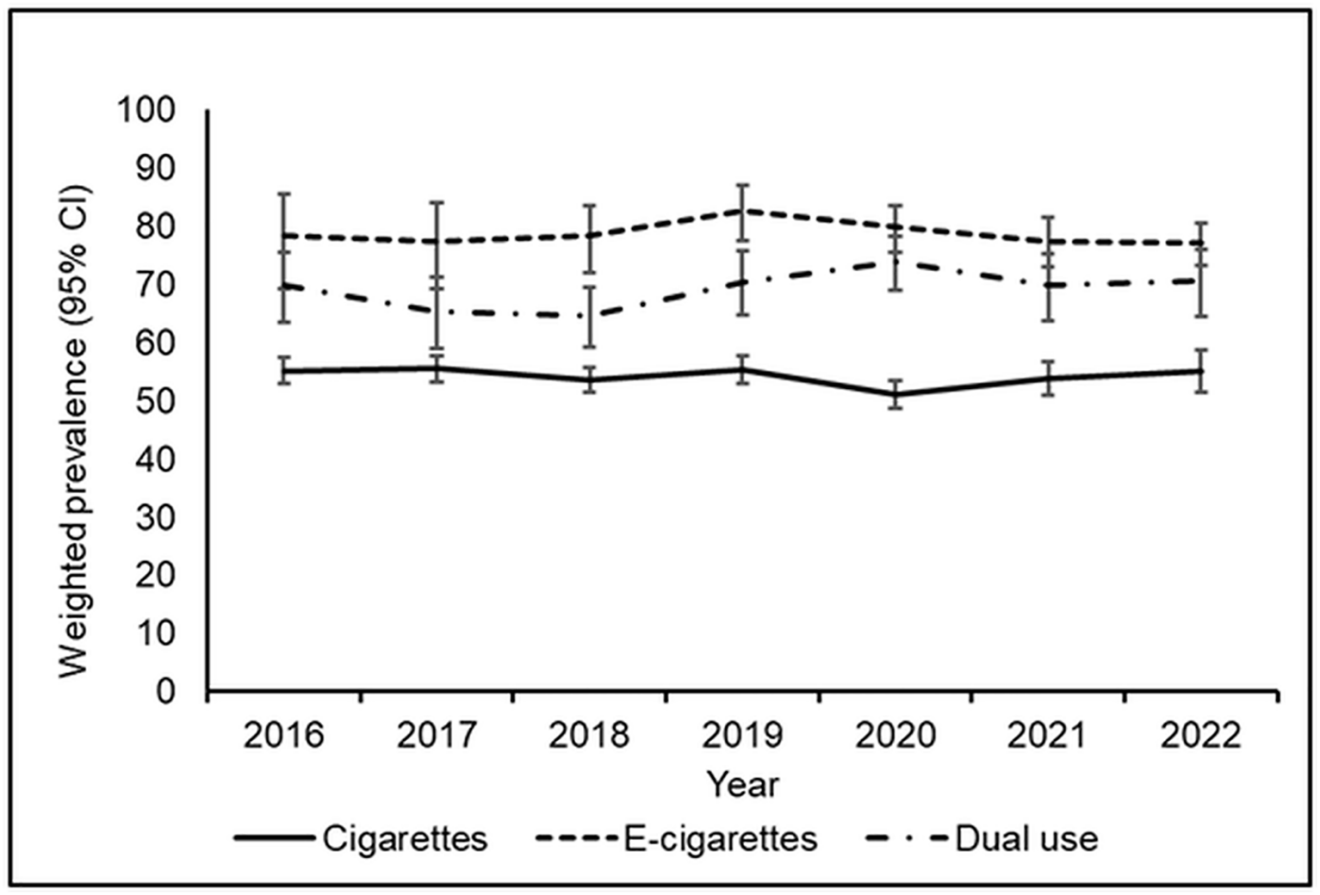
Annual rates of cigarette, e-cigarette, and dual use cessation during pregnancy (PRAMS Phase 8 data).

Minimal variation in annual cessation rates was observed from 2016 to 2022 across CC (range: 51.2%–55.5%), EC (range: 77.1%–82.8%), and dual-use (range: 64.6%–73.9%), as shown in Fig 3.

Most respondents were non-Hispanic whites aged 20‒34 from urban communities, with a high school or equivalent diploma or associate degree, as seen in S3 Table in S2 File. Tobacco users had higher Medicaid coverage, increased rates of depression, abuse history, alcohol use, and unintended pregnancies. Differences in PNC utilization were also evident, with smokers or ENDS users more likely to have irregular prenatal vitamins intake, Women, Infants, and Children (WIC) program (Special Supplemental Nutrition Program for Women, Infants, and Children) use, and delayed or infrequent PNC visits (S4 Table in S2 File).

The correlates significantly associated with increased odds of quitting CC cessation included higher educational attainment, Hispanic ethnicity, employer-based insurance, and primiparity (OR range: 1.9–4.2) are shown in S5 Table in S2 File. Compared with lighter smokers, women who smoked more than half a pack of cigarettes per day had the lowest odds of quitting CC use during pregnancy (OR: 0.2; 95%CI: 0.2–0.3). Inadequate APNCU Index also correlated with lower odds of quitting (OR: 0.5; 95%CI: 0.4–0.5). Lower educational attainment, rural residence, Medicaid insurance, WIC participation, unmarried status, unintended pregnancy, and a history of depression or abuse were also associated with reduced cessation odds (OR range: 0.5–0.6).

As presented in S6 Table in S2 File, higher education, Black race, and primiparity were independently associated with increased odds of EC cessation during pregnancy (OR range: 1.6–1.8). In contrast, daily EC use was closely associated with lower odds of quitting (OR 0.3; 95%CI: 0.2–0.4). Other correlates associated with reduced cessation odds (OR range: 0.6–0.8) included lower educational attainment, rural residence, Medicaid insurance, WIC participation, unmarried status, and a history of depression or abuse. Neither the delayed initiation of PNC (OR 0.7; 95%CI: 0.3–1.5) nor inadequate APNCU Index (OR 0.8; 95%CI: 0.6–1.1) was significantly associated with reduced EC cessation. However, attending fewer than seven PNC visits was independently associated with lower odds of quitting EC use (OR 0.7; 95%CI: 0.5–0.9).

As seen in S7 Table in S2 File, cessation behavior among dual users more closely resembled CC cessation than EC cessation. The correlates significantly associated with increased odds for dual-use cessation included younger maternal age, higher educational attainment, Hispanic ethnicity, employer-based insurance, and primiparity (OR range: 1.7–4.4). Compared with lighter smokers, women who smoked more than half a pack of cigarettes per day had the lowest odds of quitting dual use during pregnancy (OR: 0.2; 95%CI: 0.2–0.3). Whereas, the vaping frequency did not correlate with dual-use cessation (OR: 1.2; 0.9–1.5). Inadequate APNCU Index (OR: 0.4; 95%CI: 0.3–0.6) was associated with lower odds for quitting dual use.

Table 2 shows the adjusted associations between socio-demographic, behavioral, and PNC utilization factors and the odds of quitting CC, EC, and dual use during pregnancy. Primiparity (AOR range: 1.6–2.3) and Black race (AOR range: 1.4–2.1) were consistently associated with higher odds of quitting across exclusive and dual use of CC and EC, while Hispanic ethnicity was significantly associated with quitting CC and dual use (AOR range: 2.2–3.0).

**Table 2:** Adjusted sociodemographic and prenatal care utilization correlates of quitting cigarettes, e-cigarettes, and dual-use during pregnancy (PRAMS Phase 8 data).

Characteristic	Quits cigarettes		Quits e-cigarettes		Quits dual use	
	AOR^a^ [95%CI]	*p*-value	AOR^a^ [95%CI]	*p*-value	AOR^a^ [95%CI]	*p*-value
Age (years)		.001		.056		.001
<20	1.3 [1.0,1.7]		1.1 [0.7,1.8]		2.5 [1.4,4.3]	
20-34	Reference		Reference		Reference	
>34	0.8 [0.7,0.9]		0.6 [0.4,0.9]		0.7 [0.5,1.0]	
Education		<.001		.318		<.001
less than high school	0.6 [0.5,0.7]		0.7 [0.5,1.1]		0.4 [0.3,0.6]	
high school, some college, no degree	Reference		Reference		Reference	
bachelor's and above	2.1 [1.8,2.5]		1.1 [0.8,1.6]		1.8 [1.0,3.2]	
Race & Ethnicity		<.001		.161		<.001
Hispanic	2.2 [1.9,2.6]		1.0 [0.7,1.5]		3.0 [1.9,4.8]	
White	Reference		Reference		Reference	
Black	1.4 [1.2,1.6]		1.7 [1.1,2.8]		2.1 [1.2,3.5]	
Others	1.3 [1.1,1.5]		1.1 [0.7,1.8]		1.6 [1.0,2.6]	
Residence		<.001		.295		.152
Urban	Reference		Reference		Reference	
Rural	0.8 [0.7,0.9]		0.8 [0.6,1.1]		0.8 [0.6,1.1]	
Medicaid*WIC registration		<.001		.003		.006
Medicaid	0.6 [0.5,0.7]		0.5 [0.4,0.7]		0.7 [0.4,1.0]	
WIC registration	0.9 [0.8,1.0]		1.2 [0.9,1.7]		0.8 [0.6,1.1]	
Abuse*Depression		<.001		.021		.001
Abuse	0.7 [0.6,1.0]		1.1 [0.5,2.7]		0.5 [0.2,0.9]	
Depression	0.9 [0.6,1.4]		2.9 [0.9,9.9]		0.5 [0.2,1.2]	
Marital status*Pregnancy intention*Prenatal vitamins		<.001		.279		.244
Unmarried	0.7 [0.6,0.9]		0.7 [0.4,1.1]		0.5 [0.3,0.9]	
Unintended pregnancy	0.9 [0.7,1.0]		1.3 [0.9,2.0]		1.1 [0.7,1.6]	
Irregular vitamins	0.9 [0.7,1.1]		0.6 [0.4,1.1]		0.8 [0.4,1.4]	
Parity						
Primiparity	1.8 [1.6,2.0]	<.001	1.6 [1.2,2.1]	<.001	2.3 [1.7,3.1]	<.001
Multiparity	Reference		Reference		Reference	
APNCU Index		<.001		.992		.010
Inadequate	0.6 [0.5,0.7]		0.9 [0.6,1.5]		0.6 [0.4,0.9]	
Intermediate	0.9 [0.7,1.0]		1.0 [0.7,1.4]		0.7 [0.5,1.0]	
Adequate	Reference		Reference		Reference	
Adequate plus	1.0 [0.9,1.1]		1.0 [0.7,1.3]		1.0 [0.7,1.4]	
Cigarettes per day		<.001	NA			<.001
1-10	Reference				Reference	
11-20	0.3 [0.2,0.3]				0.2 [0.2,0.3]	
>20 (one pack)	0.2 [0.2,0.3]				0.3 [0.2,0.4]	
E-cigarettes per week	NA			<.001		.241
Occasional			Reference		Reference	
Some days			0.7 [0.4,1.3]		1.0 [0.7,1.5]	
Everyday			0.3 [0.2,0.5]		1.3 [1.0,1.6]	
Alcohol						
None to occasional	Reference	<.001	Reference	.350	Reference	.001
Moderate	1.4 [1.2,1.6]		0.8 [0.5,1.1]		1.8 [1.2,2.7]	
Heavy	1.1 [0.9,1.3]		0.8 [0.5,1.3]		2.0 [1.2,3.2]	

^*a*^ Reference groups for AOR calculation under each category: age: 20–34, education: high school, some college, no degree, race & ethnicity: White non-Hispanic, residence: urban, Medicaid & WIC registration: none, partner/ ex-partner abuse and depression: none, Obstetric: multiparity, married status, intended pregnancy, regular prenatal vitamins, APNCU index: adequate, cigarettes per day: half a pack or less, e-cigarettes per week: occasional, alcohol: none to occasional.

Abbreviations: AOR – adjusted odds ratio, APNCU ‒ Adequacy of Prenatal Care Utilization, CI – confidence interval, HCW ‒ Health Care Worker, PNC – Prenatal Care, WIC ‒ Women, Infants, and Children.

In contrast, older maternal age (AOR range: 0.6–0.8) and Medicaid insurance (AOR range: 0.5–0.7) were associated with lower cessation odds in all groups. High-frequency use, namely, smoking more than half a pack of cigarettes every day had the lowest odds for quitting CC (AOR: 0.3; 95%CI: 0.2–0.3) and dual use (AOR: 0.2; 95%CI: 0.2–0.3), while vaping every day had the lowest odds (AOR: 0.3; 95%CI: 0.2–0.5) for quitting EC. Additionally, an inadequate APNCU Index was significantly associated with lower cessation odds of CC (AOR: 0.6; 95%CI: 0.5–0.7) and dual use (AOR: 0.6; 95%CI: 0.4–0.9). The dual-use cessation mirrored CC cessation behavior more closely than EC cessation.

**Fig 4:**
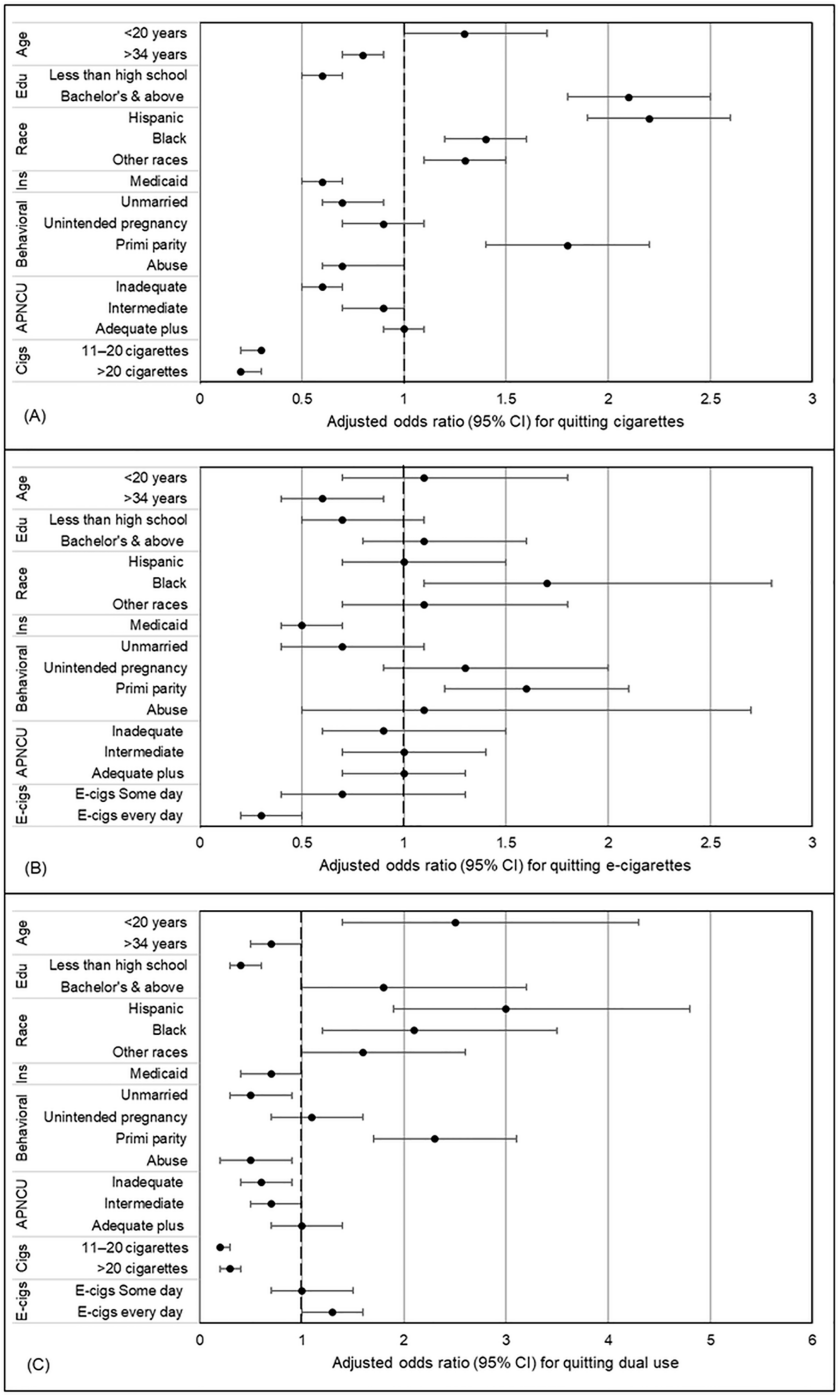
Forest plots showing adjusted odds ratios with 95% confidence intervals of the correlates of (A) cigarette cessation, (B) e-cigarette cessation, and (C) dual use cessation during pregnancy. The reference groups under each category include age: 20-34, education: high school, some college, no degree, race & ethnicity: White non-Hispanic, residence: urban, Medicaid: none, partner/ ex-partner abuse: none, APNCU index: adequate, cigarettes per day: half a pack or less, and e-cigarettes per week: occasional.

The forest plots summarize the adjusted associations presented in Table 2, allowing direct comparison of the magnitude and directions of associations across CC (Fig 4A), EC (Fig 4B), and dual-use (Fig 4C) cessation models for key socio-demographic, behavioral, and PNC variables.

Comparison of included and excluded groups to assess potential selection bias showed broadly similar socio-demographic and behavioral characteristics, and expected differences in PNC utilization, suggesting that exclusions were unlikely to substantially bias the observed cessation patterns (S8 Table in S2 File). The logistic regression models were broadly consistent in direction and magnitude across survey-year subpopulations (2016–2019); however, estimates for EC cessation were less stable due to smaller sample sizes, in contrast to the CC cessation (S9 Table in S2 File).

## Discussion

This study examined the socio-demographic factors and PNC utilization characteristics associated with CC, EC, and dual use cessation during pregnancy using PRAMS Phase 8 data. Findings indicated that a substantial proportion of women ceased CC, EC, and their dual use during pregnancy, although annual rates in quitting behavior showed minimal variation. Key factors associated with higher adjusted odds for cessation included lower pre-pregnancy exposure and primiparity. Medicaid coverage was independently linked to lower adjusted odds of quitting. An inadequate APNCI Index was associated with lower adjusted odds for CC and dual use cessations. Though an inadequate APNCU Index was not associated with EC cessation, fewer PNC visits were independently associated with lower odds of quitting EC use.

Our estimated prevalence of CC or EC use during pregnancy was lower than that reported in the PATH Study and the National Health Interview Survey (NHIS) [14,36]. According to PATH data from 2013–2014, the prevalence of CC use during pregnancy was 13.8% (95%CI: 10.5, 17.1), and EC use was 4.9% (95%CI: 3.2, 6.6) [14]. NHIS data from 2014–2017 reported CC use at 8.0% (95%CI: 3.3, 12.7) and EC use at 3.6% (95%CI: −1.1, 8.3) [36]. In contrast, our most recent data represent period prevalence among individuals who used CC or EC products before pregnancy and continued use during the third trimester. The most significant reduction in smoking prevalence among pregnant women occurred between 2000 and 2010, followed by relative stagnation between 2010 and 2020 [8,14,37]. These findings suggest gaps in PNC adequacy or HCW engagement during PNC visits, which may be relevant to ongoing tobacco use during pregnancy.

Consistent with previous research, our study showed that the pregnancy-associated cessation rates of EC users were higher than that among smokers [17,21,25,38]. We found that about 4% of CC and dual users transitioned to exclusive EC use during pregnancy, mirroring findings from PATH data [39]. Pregnant women often perceive EC as a harm reduction or smoking cessation strategy [17,40]. While prior studies have documented the correlates of continued EC and dual use during pregnancy [22,24,39,41], only a few have explored the characteristics of women who successfully quit [21,38,42].

A longitudinal analysis of PATH data (2013–2015) identified pregnancy status and being an experimental rather than established EC user as significant predictors of quitting [38], whereas externalizing rather than internalizing psychiatric conditions were associated with lower odds of cessation. Like our findings, the PATH data analysis did not find significant associations between EC cessation and maternal age, race, poverty level, or alcohol use. Our findings add to prior research on EC and dual-use cessations using part of the PRAMS Phase 8 data [21,42]. The proportion of dual users quitting both CC and EC or quitting only EC was higher than those quitting CC, but the factors associated with quitting dual use aligned more closely with CC cessation than EC cessation.

The association between higher pre-pregnancy smoking and vaping frequency and continued use during pregnancy is consistent with nicotine dependence, suggesting that different or more intensive cessation approaches may be needed for some women. On average, pregnant women who continued CC use consumed 11 cigarettes per day and smoked on 26 days per month [14]. The EC users who continued vaping did so on 13 days per month. Among pregnant women using EC, 38.4% reported using nicotine-containing products, and 26.4% were unaware of the nicotine content of their EC products [17]. Most adult daily EC users consume nicotine-containing products [43].

Similar to our findings, other studies also reported higher rates of inadequate PNC utilization among smokers and dual users compared to EC users [24,41]. The PRAMS data did not allow us to determine whether inadequate PNC utilization contributed to lower cessation rates or whether both inadequate APNCU and reduced cessation were driven by underlying factors associated with maternal characteristics. Nearly 45% of women quit smoking spontaneously before their first PNC visit [44]. Brief direct counseling interventions (5–15 minutes) provided by HCWs can lead to smoking cessation rates of 5–10%. However, around 25% of women who smoke, especially Black and Hispanic women, do not disclose their smoking status during PNC visits.

The lack of an observed association between the APNCU and EC cessation warrants careful interpretation. EC use is perceived by pregnant women as a harm-reduction strategy or a safer alternative to combustible tobacco [14–18]. Many pregnant women do not perceive ENDS as a form of tobacco or nicotine use and may be unaware of the nicotine content of these products, which are marketed with substantial variability in nicotine formulation and labeling and less tightly regulated [10,11,17]. These perceptions may attenuate motivation to discontinue EC use during pregnancy, even among women receiving timely and adequate PNC.

In addition, PNC encounters have historically prioritized screening and counseling for CC, with less consistent emphasis on ENDS [12,18]. It is possible that many EC users met the criteria for adequate PNC utilization as assessed using the APNCU, yet did not receive targeted counseling or cessation support specific to EC use, a potential limitation of the APNCU Index. This misalignment between PNC quantity and counseling content may partly explain why cessation of EC use was not significantly associated with either the HCW inquiry about smoking or the APNCU Index.

Our results suggested that Medicaid coverage by itself did not lead to effective utilization of tobacco or nicotine cessation services. Notably, presumptive Medicaid eligibility for PNC services while enrollment is pending increased smoking cessation by 7.7% [45]. However, Medicaid beneficiaries often experienced delays in initiating PNC compared to those with private insurance [46]. Despite Medicaid coverage for pharmacotherapy and counseling for smoking cessation since 2010, fewer than 1% of pregnant smokers utilized these services [38].

### Strengths and limitations

This study benefits from the use of weighted PRAMS data, which covers over 80% of U.S. women with live births [34]. PRAMS provides a large sample size and the most recent insights into EC and dual-use behaviors among pregnant women. Additionally, our study adds new insights into how PNC utilization relates to cessation patterns, particularly for EC and dual use, which remain under-studied.

However, some limitations must be acknowledged. The cross-sectional PRAMS data limit insights into the evolution of quitting behaviors or drawing causal inferences. Because PRAMS includes only women with live births, survivorship bias may result in overestimation of pregnancy-associated cessation rates and underestimation of the risks associated with continuation. Tobacco use is self-reported and collected postpartum, introducing the potential recall and social desirability bias. The PRAMS data do not distinguish between ENDS or indicate nicotine content, limiting assessment of product-specific cessation effects. PRAMS also do not provide data on household smoking, an important correlate of quitting [1,28].

Cessation was defined as discontinuation of CC and/or EC use in the last three months of pregnancy relative to the three months before pregnancy. This definition may misclassify individuals who quit in the first or second trimester but relapse by late pregnancy as continuers and may underestimate women who substantially reduced cigarette consumption without fully quitting. In addition, trimester-specific EC cessation trajectories could not be assessed due to data availability.

Collectively, these factors may lead to both over- and under-estimation of absolute cessation rates, but are unlikely to fully account for the observed differential patterns in cessation across product types.

## Conclusion

This study provides a comprehensive analysis of the socio-demographic and PNC correlates of cessation of CC, EC, and dual-use behaviors among pregnant women in the U.S. While pregnancy is associated with higher rates of tobacco product cessation, these behaviors vary significantly by socio-demographic characteristics and PNC utilization. Notably, EC cessation was not significantly associated with the APNCU Index, whereas cessation of CC and dual-use was significantly associated with PNC adequacy. Overall, considering the observed associations with age, parity, and pre-pregnancy tobacco exposure, integrating PNC settings with targeted cessation support may represent an important opportunity, particularly for high-risk groups. Furthermore, cessation strategies that address the distinct challenges posed by rising EC and dual use during pregnancy warrant further evaluation.

## Supporting information

S1 File Checklist. This file contains the STROBE checklist for cross sectional studies. (DOC)

S2 File Supporting Information. This file contains supplementary methods (S1 Table) and additional results (S2 Table–S9 Table) referenced in the main manuscript. (DOCX)
