# Supplementary material for: Tobacco product cessation and prenatal care utilization: A Pregnancy Risk Assessment Monitoring System phase 8 study of conventional cigarette, e-cigarette, and dual-use associated behaviors
Source: PLoS One. 2026 Mar 3;21(3):e0343423. doi: 10.1371/journal.pone.0343423 (PMC12956092; doi:10.1371/journal.pone.0343423)
Supplement: S2 File — (DOCX) [file pone.0343423.s002.docx]

**Manuscript:** Tobacco product cessation and prenatal care utilization: A Pregnancy Risk Assessment Monitoring System Phase 8 study of conventional cigarette, e-cigarette, and dual-use associated behaviors

**Supplemental File 2.** Supporting Information

***Data availability:*** The PRAMS Phase 8 dataset was obtained from the CDC PRAMS Analytic Research File (http://pramsarf.cdc.gov). Details of the reclassification of the analytical variables is provided in this Supporting Information File.The corresponding author can also provide a detailed data curation statement upon a reasonable request.

**S1 Table.** Details of variable reclassification.

| **Study variables** | **PRAMS coding** | **Categories** |
| --- | --- | --- |
| Year of survey | ID | 2016 to 2022 |
| Maternal age | MAT_AGE_PU (scale), MAT_AGE_NAPHSIS_AK (categorical), MAT_AGE_NAPHSIS_VT (categorical) | Numerical data and categories, namely, <20, 20-24, 25-29, 30-34, 35-39, 40+ merged into three categories: <20, 20-34, >34 |
| Education | MAT_ED (8^th^ grade and less, 9-12 grade, high school graduate-diploma, some college – no/associate degree, bachelors/ masters/ doctorate/ professor) | Reclassified as less than high school, high school-some college-no degree, and bachelor’s and above. |
| Race & ethnicity | HISP_BC, MAT_RACE_PU, MRACE_ASIAN_PU, MRACE_BLK, MRACE_MULTO_PU, MRACE_NHOPI_PU, MRACE_WHT, MRACE_AMI, MAT_RACE_PU_AK | Multiple columns were merged to create – Hispanic and non-Hispanic White, Black, and Others (Asian, Alaskan Native, Pacific Islanders, and multi-ethnicity). |
| Smoking and e-cigarette use in the three months before pregnancy and the last three months of pregnancy | SMK63B_A, SMK63L_A, ECIG_3B_A, ECIG_3L_A | Data recoded to get exclusive use of cigarettes, e-cigarettes, and their dual use before and during pregnancy, and their quitting behavior. |
| Frequency of cigarettes and e-cigarette use three months before pregnancy | SMK6_3B (>40, 21-40, 11-20, 6-10, 1-5), ECIG_3B (>1/day, 1/day, 2-6 a week, 1 day a week or less) | Smoking frequency data recategorized as 1-10, 11-20, >20  E-cigarette frequency recategorized as occasional. Some days, every day of a week. |
| Abuse during pregnancy by a partner or ex-partner | PAD6HUS, PAD_XHUS | Merged |
| Pregnancy intention | PGINTENT (Later, sooner, then, did not want, was not sure) | Sooner and then – intended; later, did not want, was not sure – unintended |
| Alcohol three months before pregnancy | DRK83B_A (none), DRK8_3B (14 or more/week, 8-13, 4-7, 1-3, <1, didn’t) | ≥8 per week (heavy), 4-7 (moderate), ≤3 or none (None to occasional) |
| Vitamins pre-pregnancy | VITAMIN | Didn’t take & 1-3 times per week – irregular; 4-6 & everyday - regular |
| First prenatal care visit | PNC_WKS (Numeric data) | Two categories: ≤16 and >16 weeks based on Intermediate APNCU Index (4^th^ month) |
| Number of prenatal care visits | PNC_VST (Numeric data) | Two categories: >6 and ≤6 based on Intermediate APNCU Index (50-79% of number of recommended visits). For a 40-week pregnancy, ACOG recommends 14 visits. |

**S2 Table.** Descriptive data on cigarette and e-cigarette use three months before and the last three months of pregnancy (PRAMS Phase 8 data).

| **Cigarette and e-cigarette use before pregnancy** | **Cigarette and e-cigarette use during pregnancy** | **Unweighted count** | **Weighted count** | **Weighted prevalence** |
| --- | --- | --- | --- | --- |
| ***n (N)*; % [95%CI]** |  | ***n*=223,793** | ***N*=11,475,844** | **% [95%CI]** |
| No cigarette or  e-cigarette  183146 (9528870);  83.1 [82.8,83.3] | No cigarette or  e-cigarette | 182,990 | 9,519,536 | 83.0 [82.7,83.2] |
|  | Cigarettes ^a^ | 114 | 6244 | 0.1 [0.0,0.1] |
|  | E-cigarettes ^b^ | 40 | 2845 | 0.0 [0.0,0.0] |
|  | Dual use ^a,b,c^ | 2 | 244 | 0.0 [0.0,0.0] |
| Cigarette  29743 (1389845);  12.1 [11.9,12.3] | Continues | 14296 | 625,296 | 5.4 [5.3,5.6] |
|  | Quits | 15012 | 741,866 | 6.5 [6.3,6.6] |
|  | E-cigarettes ^b^ | 143 | 7531 | 0.1 [0.1,0.1] |
|  | Dual use ^b,c^ | 292 | 15151 | 0.1 [0.1,0.2] |
| E-cigarette  4603 (242372);  2.1 [2.0,2.2] | Continues | 953 | 51597 | 0.4 [0.4,0.5] |
|  | Quits | 3604 | 189,510 | 1.7 [1.6,1.7] |
|  | Cigarettes ^a^ | 35 | 1510 | 0.0 [0.0,0.0] |
|  | Dual use ^a,c^ | 11 | 356 | 0.0 [0.0,0.0] |
| Dual user of cigarette and e-cigarette  6301 (314158);  2.7 [2.6,2.8] | Continues | 1422 | 67431 | 0.6 [0.5,0.6] |
|  | Quits both | 2995 | 153,107 | 1.3 [1.3,1.4] |
|  | Cigarettes only | 1424 | 70150 | 0.6 [0.6,0.7] |
|  | E-cigarettes only | 460 | 23470 | 0.2 [0.2,0.2] |

*n(N*): ^a^ new smokers ‒162 (8354), ^b^ new e-cigarette users ‒ 477 (25772), ^c^ new dual users ‒ 305 (15751).

**S3 Table.** Characteristics of tobacco product users and non-users during pregnancy (PRAMS Phase 8 data).

| **Characteristic** | **Weighted proportion**  **% [95%CI]** | | | **Missing data percent** |
| --- | --- | --- | --- | --- |
|  | **Tobacco product users^a^** | **Tobacco product non-users^a^** | **Study**  **population** |  |
| *Sociodemographic* |  |  |  |  |
| Age (years) |  |  |  | 0 |
| <20 | 5.2 [4.7,5.7] | 3.9 [3.8,4.1] | 4.0 [3.9-4.1] |  |
| 20-34 | 81.8 [80.9,82.7] | 76.5 [76.2,76.7] | 76.9 [76.6,77.1] |  |
| >34 | 13.0 [12.3,13.8] | 19.6 [19.4,19.9] | 19.1 [18.9,19.4] |  |
| Education |  |  |  | 0.8 |
| less than high school | 22.5 [21.6,23.6] | 10.3 [10.1,10.5] | 11.2 [11.0,11.4] |  |
| high school, some college | 72.2 [71.1,73.3] | 49.1 [48.7,49.4] | 50.8 [50.5,51.1] |  |
| Bachelor's and above | 5.2 [4.7,5.8] | 40.6 [40.3,40.9] | 37.9 [37.6,38.2] |  |
| Race & Ethnicity |  |  |  | 0.8 |
| Hispanic | 6.3 [5.8,6.9] | 18.6 [18.4,18.9] | 17.7 [17.5,17.9] |  |
| White | 75.4 [74.5,76.3] | 57.2 [56.9,57.5] | 58.6 [50.5,51.1] |  |
| Black | 11.6 [10.9,12.3] | 14.4 [14.2,14.6] | 14.2 [14.0,14.4] |  |
| Others | 6.6 [6.2,7.1] | 9.8 [9.6,10.0] | 9.5 [9.4,9.7] |  |
| Residence |  |  |  | 4.6 |
| Urban | 70.1 [69.1,71.2] | 85.5 [85.3,85.7] | 84.4 [84.1,84.6] |  |
| Rural | 29.9 [28.8,30.9] | 14.5 [14.3,14.7] | 15.6 [15.4,15.9] |  |
| Medical Insurance |  |  |  |  |
| Any insurance |  |  |  | 3.4 |
| Yes | 98.9 [98.7,99.1] | 97.4 [97.3,97.5] | 97.5 [97.4,97.6] |  |
| Uninsured | 1.1 [0.9,1.3] | 2.6 [2.5,2.7] | 2.5 [2.4,2.6] |  |
| Medicaid |  |  |  | 3.6 |
| Yes | 76.4 [75.3,77.4] | 37.5 [37.2,37.8] | 40.4 [40.1,40.7] |  |
| No | 23.6 [22.6,24.7] | 62.5 [62.2,62.8] | 59.6 [59.3,59.9] |  |
| Employer |  |  |  | 3.5 |
| Yes | 16.6 [15.8,17.5] | 51.2 [50.9,51.5] | 48.6 [48.3,48.9] |  |
| No | 83.4 [82.5,84.2] | 48.8 [48.5,49.1] | 51.4 [51.1,51.7] |  |
| Abuse by a partner or ex-partner |  |  |  | 2.0 |
| Yes | 7.1 [6.5,7.8] | 1.4 [1.3,1.5] | 1.8 [1.8,1.9] |  |
| No | 92.9 [92.2,93.5] | 98.6 [98.5,98.7] | 98.2 [98.1,98.2] |  |
| Depression |  |  |  | 1.0 |
| Yes | 36.9 [35.7,38.0] | 12.5 [12.2,12.7] | 14.3 [14.1,14.5] |  |
| No | 63.1 [62.0,64.3] | 87.5 [87.3,87.8] | 85.7 [85.5,85.9] |  |
| Alcohol use |  |  |  | 0.7 |
| None to occasional | 85.8 [85.0,86.7] | 89.7 [89.6,89.9] | 89.5 [89.3,89.6] |  |
| Moderate | 8.2 [7.5,8.8] | 7.8 [7.7,8.0] | 7.9 [7.7,8.0] |  |
| Heavy | 6.0 [5.4,6.6] | 2.4 [2.3.2.5] | 2.7 [2.6,2.8] |  |

**S3 Table (continued).** Characteristics of tobacco product users and non-users during pregnancy (PRAMS Phase 8 data).

| **Characteristic** | **Weighted proportion**  **% [95%CI]** | | | **Missing data percent** |
| --- | --- | --- | --- | --- |
|  | **Tobacco product users^a^** | **Tobacco product non-users^a^** | **Study**  **population** |  |
| *Obstetric* |  |  |  |  |
| Parity |  |  |  | 0.1 |
| Primi | 29.5 [28.4,30.6] | 40.0 [39.7,40.3] | 39.2 [38.9,39.5] |  |
| Multiparity | 70.5 [69.4,71.4] | 60.0 [59.7,60.3] | 60.8 [60.5,61.1] |  |
| Marital status |  |  |  | 0.1 |
| Yes | 28.2 [27.2,29.3] | 65.0 [64.7,65.4] | 62.3 [61.9,62.6] |  |
| Unmarried | 71.8 [70.7,72.8] | 35.0 [34.6,35.3] | 37.7 [37.4,38.1] |  |
| Pregnancy intention |  |  |  | 1.6 |
| Yes | 36.6 [35.5,37.8] | 62.7 [62.3,63.0] | 60.7 [60.4,61.0] |  |
| Unintended | 63.4 [62.2,64.5] | 37.3 [37.0,37.7] | 39.3 [39.0,39.6] |  |
| Medical |  |  |  |  |
| Gestational hypertension (yes) | 10.8 [10.1,11.5] | 10.1 [9.9,10.3] | 10.2 [10.0,10.4] | 0.1 |
| Gestational diabetes (yes) | 6.9 [6.4,7.6] | 7.6 [7.4,7.7] | 7.5 [7.3,7.7] | 0.1 |

^a^ cigarettes and/or e-cigarettes

**S4 Table.** Health-seeking and prenatal care utilization characteristics of tobacco product users and non-users (PRAMS Phase 8 data).

| **Characteristic** | **Weighted proportion**  **% [95%CI]** | | | **Missing data percent** |
| --- | --- | --- | --- | --- |
|  | **Tobacco product users^a^** | **Tobacco product non-users^a^** | **Study population** |  |
| Prenatal vitamins |  |  |  | 0.5 |
| Regular | 19.8 [18.9,20.7] | 45.0 [44.6,45.3] | 43.1 [42.7,43.4] |  |
| None to 3 times/ week | 80.2 [79.3,81.1] | 55.0 [54.7,55.4] | 56.9 [56.6,57.3] |  |
| WIC registration |  |  |  | 1.4 |
| Yes | 56.7 [55.5,57.9] | 30.8 [30.5,31.1] | 32.8 [32.5,33.1] |  |
| No | 43.3 [42.1,44.5] | 69.2 [68.9,69.5] | 67.2 [66.9,67.5] |  |
| HCW inquiry before pregnancy on smoking |  |  |  | 2.3 |
| Yes | 75.6 [75.3,75.9] | 90.1 [89.1,91.0] | 76.5 [76.2,76.9] |  |
| No | 24.4 [24.1,24.7] | 9.9 [9.0,10.9] | 23.5 [23.1,23.8] |  |
| HCW inquiry during pregnancy: |  |  |  |  |
| Smoking | 98.2 [97.9,98.5] | 94.4 [94.3,94.6] | 94.7 [94.6,94.9] | 2.3 |
| Alcohol | 94.6 [94.0,95.1] | 93.9 [93.7,94.1] | 94.0 [93.8,94.1] | 2.4 |
| Drugs | 87.0 [86.2,87.8] | 79.2 [78.9,79.5] | 79.8 [79.5,80.0] | 2.6 |
| Abuse | 77.1 [76.1,78.1] | 72.3 [72.0,72.6] | 72.7 [72.4,72.9] | 2.7 |
| Depression | 83.5 [82.6,84.4] | 78.8 [78.5,79.0] | 79.1 [78.9,79.4] | 2.6 |
| Plan on breastfeeding | 92.8 [92.2,93.4] | 90.1 [89.9,90.3] | 90.3 [90.1,90.5] | 2.4 |
| First PNC visit |  |  |  | 3.0 |
| ≤16 weeks | 95.5 [95.0,95.9] | 98.2 [98.2,98.3] | 98.0 [97.9,98.1] |  |
| >16 weeks | 4.5 [4.1,5.0] | 1.8 [1.7,1.8] | 2.0 [1.9,2.1] |  |
| Number of PNC visits |  |  |  | 0.2 |
| >6 | 82.2 [81.3,83.1] | 91.7 [91.5,91.9] | 91.0 [90.8,91.2] |  |
| ≤6 | 17.8 [16.9,18.7] | 8.3 [8.1,8.5] | 9.0 [8.8,9.2] |  |
| APNCU Index |  |  |  | 0.0 |
| Inadequate | 20.8 [19.9,21.8] | 11.6 [11.3,11.8] | 12.3 [12.1,12.5] |  |
| Intermediate | 11.1 [10.4,11.9] | 10.9 [10.7,11.1] | 10.9 [10.7,11.1] |  |
| Adequate | 38.4 [37.2,39.5] | 47.1 [46.8,47.5] | 46.5 [46.2,46.8] |  |
| Adequate plus | 29.7 [28.6,30.8] | 30.4 [30.1,30.7] | 30.4 [30.1,30.7] |  |

^a^ cigarettes and/or e‒cigarettes

*APNCU ‒ Adequacy of Prenatal Care Utilization, HCW ‒ Health Care Worker, PNC – Prenatal Care, WIC – Women, Infants, and Children*

**S5 Table.** Correlates of quitting conventional cigarettes during pregnancy (PRAMS Phase 8 data).

| **Characteristic** | **Weighted proportion**  **% [95%CI]** | | **Unadjusted odds ratio*^a^***  **OR [95%CI]** | ***p*-value** |
| --- | --- | --- | --- | --- |
|  | **Quits**  **cigarettes** | **Continues cigarettes** |  |  |
| *Survey year* |  |  |  |  |
| 2016 | 54.9 [52.6,57.2] | 45.1 [42.8,47.4] | Reference |  |
| 2022 | 55.0 [51.3,58.7] | 45.0 [41.3,48.7] | 1.0 [0.8,1.2] | .127 |
| *Sociodemographic* |  |  |  |  |
| Age (years) |  |  |  | <.001 |
| <20 | 4.6 [4.1,5.2] | 4.0 [3.4,4.6] | 1.2 [1.0,1.4] |  |
| 20-34 | 80.6 [79.6,81.5] | 82.1 [81.0,83.1] | Reference |  |
| >34 | 14.8 [14.0,15.7] | 14.0 [13.1,14.9] | 1.1 [1.0,1.2] |  |
| Education |  |  |  | <.001 |
| less than high school | 12.6 [11.7,13.5] | 23.4 [22.2,24.6] | 0.6 [0.5,0.6] |  |
| high school, some college, no degree | 68.1 [66.9,69.3] | 71.7 [70.4,73.0] | Reference |  |
| Bachelor's and above | 19.3 [18.4,20.3] | 4.9 [4.3,5.5] | 4.2 [3.6,4.8] |  |
| Race & Ethnicity |  |  |  | <.001 |
| Hispanic | 12.2 [11.5,13.0] | 6.1 [5.5,6.7] | 2.2 [2.0,2.6] |  |
| White | 65.7 [64.6,66.8] | 73.4 [72.3,74.5] | Reference |  |
| Black | 14.9 [14.0,15.7] | 13.8 [13.0,14.7] | 1.2 [1.1,1.3] |  |
| Others | 7.2 [6.7,7.8] | 6.7 [6.1,7.3] | 1.2 [1.1,1.4] |  |
| Residence |  |  |  |  |
| Urban | 79.5 [78.5,80.5] | 70.2 [68.9,71.4] | Reference | <.001 |
| Rural | 20.5 [19.5,21.5] | 29.8 [28.6,31.1] | 0.6 [0.6,0.7] |  |
| Medical Insurance |  |  |  |  |
| Any insurance |  |  |  |  |
| Yes | 98.7 [98.4,99.0] | 99.0 [98.8,99.2] | Reference |  |
| Uninsured | 1.3 [1.0,1.6] | 1.0 [0.8,1.2] | 1.0 [0.7,1.5] | .830 |
| Medicaid |  |  |  |  |
| Yes | 56.7 [55.5,58.0] | 77.0 [75.7,78.1] | 0.6 [0.5,0.6] | <.001 |
| No | 43.3 [42.0,44.5] | 23.0 [21.9,24.3] | Reference |  |
| Employer |  |  |  |  |
| Yes | 35.6 [34.4,36.9] | 16.7 [15.7,17.8] | 1.9 [1.7,2.1] | <.001 |
| No | 64.4 [63.1,65.6] | 83.3 [82.2,84.3] | Reference |  |

*Table continued on the next page*

**S5 Table (continued).** Correlates of quitting conventional cigarettes during pregnancy (PRAMS Phase 8 data).

| **Characteristic** | **Weighted proportion**  **% [95%CI]** | | **Unadjusted odds ratio*^a^***  **OR [95%CI]** | ***p*-value** |
| --- | --- | --- | --- | --- |
|  | **Quits**  **cigarettes** | **Continues cigarettes** |  |  |
| *Behavioral* |  |  |  |  |
| Abuse (partner/ ex-partner) |  |  |  |  |
| Yes | 3.4 [2.9,3.9] | 6.9 [6.2,7.6] | 0.5 [0.4,0.6] | <.001 |
| No | 96.6 [96.1,97.1] | 93.1 [92.4,93.8] | Reference |  |
| Depression |  |  |  |  |
| Yes | 22.0 [21.0,23.1] | 34.6 [33.3,36.0] | 0.5 [0.5,0.6] | <.001 |
| No | 78.0 [76.9,79.0] | 65.4 [64.0,66.7] | Reference |  |
| Cigarettes per day |  |  |  |  |
| 1-10 | 83.5 [82.5,84.4] | 52.4 [51.0,53.8] | Reference | <.001 |
| 11-20 | 13.3 [12.4,14.2] | 35.9 [34.5,37.2] | 0.2 [0.2,0.3] |  |
| >20 (one pack) | 3.2 [2.8,3.7] | 11.7 [10.8,12.6] | 0.2 [0.1,0.2] |  |
| Alcohol use |  |  |  | <.001 |
| None to occasional | 79.8 [78.8,80.8] | 86.8 [85.8,87.7] | Reference |  |
| Moderate | 13.5 [12.6,14.3] | 7.6 [6.9,8.4] | 1.9 [1.7,2.2] |  |
| Heavy | 6.8 [6.2,7.4] | 5.6 [5.0,6.3] | 1.3 [1.1,1.5] |  |
| *Obstetric & PNC* |  |  |  |  |
| Parity |  |  |  |  |
| Primiparity | 41.8 [40.5,43.0] | 25.6 [24.5,26.9] | 2.1 [1.9,2.3] | <.001 |
| Multiparity | 58.2 [57.0,59.5] | 74.4 [73.1,75.5] | Reference |  |
| Married | 41.7 [40.5,43.0] | 28.3 [27.1,29.5] | Reference |  |
| Unmarried | 58.3 [57.0,59.5] | 71.7 [70.5,72.9] | 0.6 [0.5,0.6] | <.001 |
| Intended pregnancy | 49.0 [47.7,50.2] | 36.5 [35.1,37.8] | Reference |  |
| Unintended pregnancy | 51.0 [49.8,52.3] | 63.5 [62.2,64.9] | 0.6 [0.6,0.6] | <.001 |
| Prenatal vitamins |  |  |  |  |
| Regular | 28.3 [27.1,29.4] | 20.1 [19.0,21.2] | Reference |  |
| None to 3 times/ week | 71.7 [70.6,72.9] | 79.9 [78.8,81.0] | 0.6 [0.6,0.7] | <.001 |
| WIC registration | 43.1 [41.8,44.3] | 57.6 [56.2,58.9] | 0.6 [0.5,0.6] | <.001 |
| No | 56.9 [55.7,58.2] | 42.4 [41.1,43.8] | Reference |  |
| HCW inquiry on smoking | 97.1 [96.7,97.5] | 98.6 [98.2,98.8] | Reference |  |
| No | 2.9 [2.5,3.3] | 1.4 [1.2,1.8] | 2.0 [1.6,2.6] | <.001 |

*Table continued on the next page*

**S5 Table (continued).** Correlates of quitting conventional cigarettes during pregnancy (PRAMS Phase 8 data).

| **Characteristic** | **Weighted proportion**  **% [95%CI]** | | **Unadjusted odds ratio*^a^***  **OR [95%CI]** | ***p*-value** |
| --- | --- | --- | --- | --- |
|  | **Quits**  **cigarettes** | **Continues cigarettes** |  |  |
| Gestational hypertension | 11.7 [11.0,12.5] | 10.9 [10.1,11.8] | 1.1 [1.0,1.2] | .171 |
| Gestational diabetes | 7.8 [7.1,8.5] | 7.3 [6.6,8.1] | 1.1 [0.9,1.2] | .365 |
| First PNC visit |  |  |  |  |
| ≤16 weeks | 97.8 [97.4,98.1] | 95.6 [95.0,96.1] | Reference |  |
| >16 weeks | 2.2 [1.9,2.6] | 4.4 [3.9,5.0] | 0.5 [0.4,0.6] | <.001 |
| PNC visits |  |  |  |  |
| >6 | 91.2 [90.5,91.9] | 81.5 [80.5,82.6] | Reference |  |
| ≤6 | 8.8 [8.1,9.5] | 18.5 [17.4,19.5] | 0.4 [0.4,0.5] | <.001 |
| APNCU Index |  |  |  | <.001 |
| Inadequate | 12.0 [11.2,12.8] | 21.1 [20.0,22.2] | 0.5 [0.4,0.5] |  |
| Intermediate | 10.5 [9.8,11.4] | 11.2 [10.4,12.1] | 0.8 [0.7,0.9] |  |
| Adequate | 44.4 [43.2,45.7] | 38.0 [36.7,39.4] | Reference |  |
| Adequate plus | 33.0 [31.8,34.2] | 29.7 [28.4,31.0] | 1.0 [0.9,1.0] |  |

^a^ Reference groups for OR calculation under each category: survey year: 2016, age: 20-34, education: high school, some college, no degree, race & ethnicity: White non-Hispanic, residence: urban, medical insurance: uninsured, no Medicaid, no employer insurance, behavioral: no partner/ ex-partner abuse, no depression, none to occasional alcohol use, cigarettes per day: half a pack or less, Obstetric: multiparity, married status, intended pregnancy, regular prenatal vitamins, no WIC registration, HCW inquired on smoking, no gestational hypertension, no gestational diabetes, PNC ≤16 weeks, >6 PNC visits, adequate APNCU index.

Abbreviations: APNCU ‒ Adequacy of Prenatal Care Utilization, CI – Confidence Interval, HCW ‒ Health Care Worker, OR – Odds Ratio, PNC – Prenatal Care, WIC ‒ Women, Infants, and Children.

**S6 Table.** Correlates of quitting e-cigarettes during pregnancy (PRAMS Phase 8 data).

| **Characteristic** | **Weighted proportion**  **% [95%CI]** | | **Unadjusted odds ratio*^a^***  **OR [95%CI]** | ***p*-value** |
| --- | --- | --- | --- | --- |
|  | **Quits**  **e-cigarettes** | **Continues**  **e-cigarettes** |  |  |
| *Survey year* |  |  |  |  |
| 2016 | 78.4 [69.3,85.4] | 21.6 [14.6,30.7] | Reference |  |
| 2022 | 76.9 [73.1,80.4] | 23.1 [19.6,26.9] | 0.9 [0.5,1.5] | .696 |
| *Sociodemographic* |  |  |  |  |
| Age (years) |  |  |  | .223 |
| <20 | 11.2 [9.7,13.0] | 10.1 [7.5,13.4] | 1.1 [0.8,1.6] |  |
| 20-34 | 80.3 [78.2,82.3] | 78.9 [74.7,82.6] | Reference |  |
| >34 | 8.4 [7.1,10.0] | 11.0 [8.4,14.3] | 0.8 [0.5,1.1] |  |
| Education |  |  |  | .001 |
| less than high school | 9.3 [7.9,10.8] | 14.3 [11.0,18.4] | 0.7 [0.5,0.9] |  |
| high school, some college, no degree | 72.4 [70.1,74.6] | 74.1 [69.4,78.3] | Reference |  |
| Bachelor's and above | 18.3 [16.4,20.3] | 11.6 [8.8,15.2] | 1.6 [1.2,2.3] |  |
| Race & Ethnicity |  |  |  | .056 |
| Hispanic | 13.6 [12.2,15.1] | 11.9 [9.0,15.5] | 1.2 [0.9,1.7] |  |
| White | 69.0 [66.9,71.0] | 74.6 [70.3,78.5] | Reference |  |
| Black | 9.5 [8.2, 11.0] | 5.8 [4.0,8.4] | 1.8 [1.2,2.7] |  |
| Others | 7.6 [5.7,10.2] | 7.6 [5.7,10.2] | 1.1 [0.8,1.6] |  |
| Residence |  |  |  |  |
| Urban | 82.7 [80.9,84.3] | 77.9 [73.7,81.6] | Reference | .024 |
| Rural | 17.3 [15.7,19.1] | 22.1 [18.4,26.3] | 0.7 [0.6,1.0] |  |
| Medical Insurance |  |  |  |  |
| Any insurance |  |  |  |  |
| Yes | 98.7 [97.8,99.2] | 98.7 [97.5,99.3] | Reference |  |
| Uninsured | 1.3 [0.8,2.2] | 1.3 [0.7,2.5] | 0.8 [0.3,1.9] | .610 |
| Medicaid |  |  |  |  |
| Yes | 52.3 [49.7,54.8] | 66.9 [62.1,71.3] | 0.6 [0.5,0.8] | .001 |
| No | 47.7 [45.2,50.3] | 33.1 [28.7,37.9] | Reference |  |
| Employer |  |  |  |  |
| Yes | 34.6 [32.2,37.1] | 23.3 [19.5,27.7] | 1.3 [0.9,1.8] | .138 |
| No | 65.4 [62.9,67.8] | 76.7 [72.3,80.5] | Reference |  |

*Table continued on the next page*

**S6 Table (continued).** Correlates of quitting e-cigarettes during pregnancy (PRAMS Phase 8 data).

| **Characteristic** | **Weighted proportion**  **% [95%CI]** | | | | **Unadjusted odds ratio*^a^***  **OR [95%CI]** | ***p*-value** |
| --- | --- | --- | --- | --- | --- | --- |
|  | **Quits**  **e-cigarettes** | | **Continues**  **e-cigarettes** | |  |  |
| *Behavioral* |  | |  | |  |  |
| Abuse (partner/ ex-partner) |  | |  | |  |  |
| Yes | 2.4 [1.8,3.1] | | 5.0 [3.1,7.7] | | 0.5 [0.3,0.8] | .007 |
| No | 97.6 [96.9,98.2] | | 95.0 [92.3,96.9] | | Reference |  |
| Depression |  | |  | |  |  |
| Yes | 27.6 [25.4,29.8] | | 36.6 [31.9,41.5] | | 0.7 [0.5,0.8] | .001 |
| No | 72.4 [70.2,74.6] | | 63.4 [58.5,68.1] | | Reference |  |
| E-cigarettes per week |  |  | |  | | <.001 |
| Occasional | 26.1 [24.0,28.3] | 11.0 [8.6,14.0] | | Reference | |  |
| Some days | 6.2 [4.2,9.0] | 10.5 [9.1,12.2] | | 0.7 [0.4,1.2] | |  |
| Every day | 63.4 [61.0,65.8] | 82.9 [79.1,86.1] | | 0.3 [0.2,0.4] | |  |
| Alcohol use |  |  | |  | |  |
| None to occasional | 82.4 [80.5,84.1] | 82.9 [78.9,86.2] | | Reference | | .967 |
| Moderate | 12.5 [11.0,14.2] | 12.0 [9.0,15.8] | | 1.0 [0.7,1.5] | |  |
| Heavy | 5.1 [4.2,6.2] | 5.2 [3.6,7.3] | | 1.0 [0.7,1.5] | |  |
| *Obstetric & PNC* |  |  | |  | |  |
| Parity |  |  | |  | | <.001 |
| Primiparity | 60.2 [57.7,62.7] | 45.2 [40.3,50.2] | | 1.8 [1.5,2.3] | |  |
| Multiparity | 39.8 [37.3,42.3] | 54.8 [49.8,59.7] | | Reference | |  |
| Married | 40.2 [37.7,42.7] | 34.0 [29.5,38.7] | | Reference | |  |
| Unmarried | 59.8 [57.3,62.3] | 66.0 [61.3,70.5] | | 0.8 [0.6,1.0] | | .024 |
| Intended pregnancy | 46.5 [44.0,49.1] | 42.9 [38.1,47.9] | | Reference | |  |
| Unintended pregnancy | 53.5 [50.9,56.0] | 57.1 [52.1,61.9] | | 0.9 [0.7,1.1] | | .203 |
| Prenatal vitamins |  |  | |  | |  |
| Regular | 29.5 [27.2,31.8] | 21.7 [18.1,25.9] | | Reference | |  |
| None to 3 times/ week | 70.5 [68.2,72.8] | 78.3 [74.1,81.9] | | 0.7 [0.5,0.9] | | .002 |
| WIC registration | 35.6 [33.3,38.0] | 41.9 [37.3,46.8] | | 0.8 [0.6,1.0] | | .019 |
| No | 64.4 [62.0,66.7] | 58.1 [53.2,62.7] | | Reference | |  |
| HCW inquiry on smoking |  |  | |  | |  |
| Yes | 96.3 [95.2,97.2] | 95.6 [92.6,97.4] | | Reference | |  |
| No | 3.7 [2.8,4.8] | 4.4 [2.6,7.7] | | 0.8 [0.4,1.5] | | .537 |

*Table continued on the next page*

**S6 Table (continued).** Correlates of quitting e-cigarettes during pregnancy (PRAMS Phase 8 data).

| **Characteristic** | **Weighted proportion**  **% [95%CI]** | | | | **Unadjusted odds ratio*^a^***  **OR [95%CI]** | ***p*-value** |
| --- | --- | --- | --- | --- | --- | --- |
|  | **Quits**  **e-cigarettes** | | **Continues**  **e-cigarettes** | |  |  |
| Gestational hypertension | 12.9 [11.3,14.7] | 8.5 [6.4,11.2] | | 1.6 [1.1,2.2] | | .007 |
| Gestational diabetes | 7.1 [5.9,8.5] | 6.0 [4.2,8.5] | | 1.2 [0.8,1.8] | | .408 |
| First PNC visit |  |  | |  | |  |
| ≤16 weeks | 98.1 [97.3,98.7] | 97.2 [94.3,98.6] | | Reference | | .339 |
| >16 weeks | 1.9 [1.3,2.7] | 2.8 [1.4,5.7] | | 0.7 [0.3,1.5] | |  |
| PNC visits |  |  | |  | |  |
| >6 | 91.6 [90.2,92.9] | 87.7 [83.9,90.7] | | Reference | | .021 |
| ≤6 | 8.4 [7.1,9.8] | 12.3 [9.3,16.1] | | 0.7 [0.5,0.9] | |  |
| APNCU Index |  |  | |  | | .681 |
| Inadequate | 11.8 [10.1, 13.6] | 14.0 [10.8,18.1] | | 0.8 [0.6,1.1] | |  |
| Intermediate | 10.3 [8.9,11.8] | 10.5 [7.9,13.7] | | 0.9 [0.6,1.3] | |  |
| Adequate | 46.5 [44.0, 49.0] | 44.3 [39.5, 49.2] | | Reference | |  |
| Adequate plus | 31.5 [29.2,33.9] | 31.2 [26.8,35.9] | | 1.0 [0.7,1.2] | |  |

^a^ Reference groups for OR calculation under each category: survey year: 2016, age: 20-34, education: high school, some college, no degree, race & ethnicity: White non-Hispanic, residence: urban, medical insurance: uninsured, no Medicaid, no employer insurance, behavioral: no partner/ ex-partner abuse, no depression, none to occasional alcohol use, e-cigarettes per week: occasional, Obstetric: multiparity, married status, intended pregnancy, regular prenatal vitamins, no WIC registration, HCW inquired on smoking, no gestational hypertension, no gestational diabetes, PNC ≤16 weeks, >6 PNC visits, adequate APNCU index.

Abbreviations: APNCU ‒ Adequacy of Prenatal Care Utilization, CI – Confidence Interval, HCW ‒ Health Care Worker, OR – Odds Ratio, PNC – Prenatal Care, WIC ‒ Women, Infants, and Children.

**S7 Table.** Correlates of quitting dual use of cigarettes and e-cigarettes during pregnancy

(PRAMS Phase 8 data).

| **Characteristic** | **Weighted proportion**  **% [95%CI]** | | **Unadjusted odds ratio*^a^***  **OR [95%CI]** | ***p*-value** |
| --- | --- | --- | --- | --- |
|  | **Quits**  **dual use** | **Continues**  **dual use** |  |  |
| *Survey year* |  |  |  |  |
| 2016 | 12.4 [11.5,13.5] | 12.1 [10.0,14.6] | Reference |  |
| 2022 | 12.3 [11.4,13.4] | 11.7 [9.7,14.1] | 1.0 [0.7,1.5] | .248 |
| *Sociodemographic* |  |  |  |  |
| Age (years) |  |  |  | <.001 |
| <20 | 12.1 [10.3,14.0] | 6.3 [4.6,8.5] | 2.0 [1.4,2.9] |  |
| 20-34 | 79.8 [77.5,81.9] | 82.7 [79.4,85.6] | Reference |  |
| >34 | 8.1 [6.9,9.6] | 11.0 [8.8,13.8] | 0.8 [0.6,1.1] |  |
| Education |  |  |  | <.001 |
| less than high school | 10.7 [9.1,12.6] | 22.8 [19.3,26.6] | 0.5 [0.4,0.6] |  |
| high school, some college, no degree | 74.0 [71.5,76.4] | 73.8 [69.8.77.4] | Reference |  |
| Bachelor's and above | 15.3 [13.4,17.3] | 3.4 [2.3,5.2] | 4.4 [2.8,6.9] |  |
| Race & Ethnicity |  |  |  | <.001 |
| Hispanic | 12.7 [11.3,14.2] | 5.4 [4.0,7.3] | 2.7 [1.9,3.9] |  |
| White | 74.1 [72.0,76.1] | 84.6 [81.7,87.1] | Reference |  |
| Black | 5.2 [4.4,6.3] | 4.8 [3.4,6.8] | 1.3 [0.8,1.9] |  |
| Others | 8.0 [6.8,9.4] | 5.2 [4.0,6.8] | 1.8 [1.2,2.5] |  |
| Residence |  |  |  | <.001 |
| Urban | 77.4 [75.1,79.5] | 65.2 [61.0,69.2] | Reference |  |
| Rural | 22.6 [20.5,24.9] | 34.8 [30.8,39.0] | 0.5 [0.4,0.7] |  |
| Medical Insurance |  |  |  |  |
| Any insurance |  |  |  |  |
| Yes | 98.7 [97.6,99.3] | 98.9 [97.8,99.4] | Reference |  |
| Uninsured | 1.3 [0.7,2.4] | 1.1 [0.6,2.2] | 0.8 [0.3,1.9] | .580 |
| Medicaid |  |  |  |  |
| Yes | 56.5 [53.7,59.3] | 80.6 [76.9,83.8] | 0.4 [0.3,0.6] | <.001 |
| No | 43.5 [40.7,46.3] | 19.4 [16.2,23.1] | Reference |  |
| Employer |  |  |  |  |
| Yes | 29.4 [27.0,31.9] | 11.8 [9.4,14.6] | 1.7 [1.2,2.5] | .001 |
| No | 70.6 [68.1,73.0] | 88.2 [85.4,90.6] | Reference |  |

*Table continued on the next page*

**S7 Table (continued).** Correlates of quitting dual use of cigarettes and e-cigarettes during pregnancy (PRAMS Phase 8 data).

| **Characteristic** | **Weighted proportion**  **% [95%CI]** | | **Unadjusted odds ratio*^a^***  **OR [95%CI]** | ***p*-value** | |
| --- | --- | --- | --- | --- | --- |
|  | **Quits**  **dual use** | **Continues**  **dual use** |  |  |  |
| *Behavioral* |  |  |  |  | |
| Abuse |  |  |  |  | |
| Yes | 4.6 [3.6,5.8] | 9.5 [7.4,12.3] | 0.5 [0.3,0.7] | <.001 | |
| No | 95.4 [94.2,96.4] | 90.5 [87.7,92.6] | Reference |  | |
| Depression |  |  |  |  | |
| Yes | 33.9 [31.2,36.6] | 48.7 [44.4,52.9] | 0.5 [0.4,0.7] | <.001 | |
| No | 66.1 [63.4,68.8] | 51.3 [47.1,55.6] | Reference |  | |
| Cigarettes per day |  |  |  | <.001 |  |
| 1-10 | 81.5 [79.2,83.7] | 47.0 [42.8,51.2] | Reference |  |  |
| 11-20 | 13.8 [12.0,15.9] | 39.0 [34.8,43.3] | 0.2 [0.2,0.3] |  |  |
| >20 (one pack) | 4.6 [3.5,6.1] | 14.1 [11.5,17.1] | 0.2 [0.1,0.3] |  |  |
| E-cigarettes per week |  |  |  | .067 |  |
| Occasional | 37.8 [35.2,40.5] | 39.4 [35.3,43.7] | Reference |  |  |
| Some days | 12.5 [10.8,14.5] | 15.9 [13.1,19.2] | 0.8 [0.6,1.1] |  |  |
| Every day | 49.6 [46.8,52.4] | 44.6 [40.5,48.9] | 1.2 [0.9,1.5] |  |  |
| Alcohol use |  |  |  | <.001 |  |
| None to occasional | 69.7 [67.0,72.2] | 86.1 [83.1,88.6] | Reference |  |  |
| Moderate | 18.4 [16.4,20.7] | 7.9 [6.0,10.5] | 2.9 [2.0,4.0] |  |  |
| Heavy | 11.9 [10.1,13.8] | 6.0 [4.5,7.9] | 2.5 [1.7,3.5] |  |  |
| *Obstetrics & PNC* |  |  |  |  |  |
| Parity | 59.9 [57.1,62.5] | 32.9 [28.9,37.1] | 3.0 [2.4,3.8] | <.001 |  |
| Primiparity |  |  |  |  |  |
| Multiparity | 40.1 [37.5,42.9] | 67.1 [62.9,71.1] | Reference | <.001 |  |
| Married | 35.9 [33.3,38.6] | 25.7 [22.4,29.4] | Reference |  |  |
| Unmarried | 64.1 [61.4,66.7] | 74.3 [70.6,77.6] | 0.6 [0.5,0.8] | <.001 |  |
| Intended pregnancy | 43.3 [40.5,46.1] | 32.3 [28.4,36.5] | Reference |  |  |
| Unintended pregnancy | 56.7 [53.9,59.5] | 67.7 [63.5,71.6] | 0.6 [0.5,0.8] | <.001 |  |
| Prenatal vitamins |  |  |  |  |  |
| Regular | 23.4 [21.2,25.9] | 19.1 [16.0,22.6] | Reference |  |  |
| None to 3 times/ week | 76.6 [74.1,78.8] | 80.9 [77.4,84.0] | 0.8 [0.6,1.0] | .042 |  |
| WIC registration | 42.0 [39.4,44.7] | 58.2 [54.0,62.4] | 0.5 [0.4,0.6] | <.001 |  |
| No | 58.0 [55.3,60.6] | 41.8 [37.6,46.0] | Reference |  |  |

*Table continued on the next page*

**S7 Table (continued).** Correlates of quitting dual use of cigarettes and e-cigarettes during pregnancy (PRAMS Phase 8 data).

| **Characteristic** | **Weighted proportion**  **% [95%CI]** | | **Unadjusted odds ratio*^a^***  **OR [95%CI]** | ***p*-value** | |
| --- | --- | --- | --- | --- | --- |
|  | **Quits**  **dual use** | **Continues**  **dual use** |  |  |  |
| HCW inquiry on smoking |  |  |  |  |  |
| Yes | 3.3 [2.4,4.4] | 2.8 [1.6,4.9] | 1.2 [0.6,2.2] | .648 |  |
| No | 96.7 [95.6,97.6] | 97.2 [95.1,98.4] | Reference |  |  |
| Gestational hypertension | 11.4 [9.8,13.3] | 10.6 [8.3,13.4] | 1.1 [0.8,1.5] | .587 |  |
| Gestational diabetes | 7.7 [6.4,9.4] | 5.0 [3.4,7.1] | 1.6 [1.0,2.5] | .035 |  |
| First PNC visit: |  |  |  |  |  |
| ≤16 weeks | 97.4 [96.1,98.3] | 93.8 [91.1,95.7] | Reference |  |  |
| >16 weeks | 2.6 [1.7,3.9] | 6.2 [4.3,8.9] | 0.4 [0.2,0.7] | .001 |  |
| PNC visits: |  |  |  |  |  |
| >6 | 92.4 [90.8,93.7] | 78.4 [74.6,81.8] | Reference |  |  |
| ≤6 | 7.6 [6.3,9.2] | 21.6 [18.2,25.4] | 0.3 [0.2,0.4] | <.001 |  |
| APNCU Index |  |  |  | <.001 |  |
| Inadequate | 13.5 [11.6,15.7] | 24.4 [20.9,28.3] | 0.4 [0.3,0.6] |  |  |
| Intermediate | 10.5 [8.8,12.3] | 12.8 [10.1,16.1] | 0.6 [0.5,0.9] |  |  |
| Adequate | 45.0 [42.2,47.8] | 35.6 [31.7,39.8] | Reference |  |  |
| Adequate plus | 31.1 [28.6, 33.6] | 27.1 [23.7, 30.9] | 0.9 [0.7,1.2] |  |  |

^a^ Reference groups for OR calculation under each category: survey year: 2016, age: 20-34, education: high school, some college, no degree, race & ethnicity: White non-Hispanic, residence: urban, medical insurance: uninsured, no Medicaid, no employer insurance, behavioral: no partner/ ex-partner abuse, no depression, none to occasional alcohol use, cigarettes per day: half a pack or less, e-cigarettes per week: occasional, Obstetric: multiparity, married status, intended pregnancy, regular prenatal vitamins, no WIC registration, HCW inquired on smoking, no gestational hypertension, no gestational diabetes, PNC ≤16 weeks, >6 PNC visits, adequate APNCU index.

Abbreviations: APNCU ‒ Adequacy of Prenatal Care Utilization, CI – Confidence Interval, HCW ‒ Health Care Worker, OR – Odds Ratio, PNC – Prenatal Care, WIC ‒ Women, Infants, and Children.

**S8 Table.** Sociodemographic, behavioral, and prenatal care utilization characteristics of women included and excluded in the study.

| **Characteristic** | **Weighted proportion**  **% [95%CI]** | |
| --- | --- | --- |
|  | **Included data**  **223,793/ 11,475,844** | **Excluded data*^a^***  **26,177/ 799,438** |
| Age (years) |  |  |
| <20 | 4.0 [3.9,4.1] | 3.9 [3.5,4.4] |
| 20-34 | 76.9 [76.6,77.1] | 74.3 [73.3,75.3] |
| >34 | 19.1 [18.9,19.4] | 21.8 [20.9,22.7] |
| Education |  |  |
| less than high school | 11.2 [11.0, 11.4] | 13.5 [12.8, 14.4] |
| high school, some college | 50.8 [50.5, 51.1] | 51.2 [50.1, 52.4] |
| bachelor's and above | 37.9 [37.6,38.2] | 35.2 [34.2,36.3] |
| Race & Ethnicity |  |  |
| Hispanic | 17.7 [17.5,17.9] | 18.8 [17.9,19.8] |
| White | 58.6 [58.3,58.8] | 52.6 [51.4,53.7] |
| Black | 14.2 [14.0,14.4] | 18.3 [17.4,19.2] |
| Others | 9.5 [9.4,9.7] | 10.3 [9.7,11.0] |
| Residence |  |  |
| Urban | 84.4 [84.1,84.6] | 85.0 [84.2,85.7] |
| Rural | 15.6 [15.4,15.9] | 15.0 [14.3,15.8] |
| Medicaid | 40.4 [40.1,40.7] | 42.2 [41.1,43.4] |
| No insurance | 2.5 [2.4,2.6] | 2.2 [1.8,2.5] |
| Employer insurance | 48.6 [48.3,48.9] | 44.6 [43.5,45.7] |
| WIC registration | 32.8 [32.5,33.1] | 36.6 [35.5,37.7] |
| Married | 62.3 [61.9,62.6] | 59.2 [58.0,60.3] |
| Intended pregnancy | 60.7 [60.4,61.0] | 59.2 [58.0,60.3] |
| Parity |  |  |
| Primi | 39.2 [38.9,39.5] | 35.5 [34.4,36.6] |
| Multi | 60.8 [60.5,61.1] | 64.5 [63.4,65.6] |
| APNCU Index |  |  |
| Inadequate | 12.3 [12.1,12.5] | 12.2 [11.3,13.2] |
| Intermediate | 10.9 [10.7,11.1] | 8.5 [7.7,9.3] |
| Adequate | 46.5 [46.2,46.8] | 33.0 [31.7,34.3] |
| Adequate plus | 30.4 [30.1,30.7] | 46.3 [45.0,47.7] |
| Abuse by a partner or ex-partner | 1.8 [1.8,1.9] | 2.2 [1.9,2.7] |
| Depression | 14.3 [14.1,14.5] | 16.6 [15.7,17.6] |
| Alcohol use |  |  |
| None to occasional | 89.5 [89.3,89.6] | 89.1 [88.3,89.8] |
| Moderate | 7.9 [7.7,8.0] | 7.8 [7.2,8.4] |
| Heavy | 2.7 [2.6,2.8] | 3.1 [2.7,3.6] |

^a^ Mothers with multiple births, missing information on parity, cigarette use, and e-cigarette use were excluded

**S9 Table.** Logistic regression model examining correlates of quitting conventional cigarettes, e-cigarettes, and dual-use during pregnancy, stratified by survey-year (2016–2019).

| **Characteristic** | **Quits cigarettes** | | **Quits e-cigarettes** | | **Quits dual use** | |
| --- | --- | --- | --- | --- | --- | --- |
|  | **AOR*^a^* [95%CI]** | ***p*-value** | **AOR*^a^* [95%CI]** | ***p*-value** | **AOR*^a^* [95%CI]** | ***p*-value** |
| Age (years) |  | .106 |  | .177 |  | .266 |
| <20 | 1.2 [0.9,1.6] |  | 0.8 [0.3,1.8] |  | 1.7 [0.9,3.5] |  |
| 20-34 | Reference |  | Reference |  | Reference |  |
| >34 | 0.9 [0.7,1.0] |  | 0.5 [0.3,1.1] |  | 0.9 [0.5,1.5] |  |
| Education |  | <.001 |  | .439 |  | <.001 |
| less than high school | 0.7 [0.6,0.8] |  | 1.5 [0.6,3.5] |  | 0.4 [0.3,0.7] |  |
| high school, some college, no degree | Reference |  | Reference |  | Reference |  |
| bachelor's and above | 2.1 [1.7,2.6] |  | 0.8 [0.4,1.4] |  | 2.2 [1.0,5.3] |  |
| Race & Ethnicity |  | <.001 |  | .180 |  | .001 |
| Hispanic | 2.4 [2.0,3.0] |  | 0.5 [0.3,1.0] |  | 2.6 [1.9,4.5] |  |
| White | Reference |  | Reference |  | Reference |  |
| Black | 1.4 [1.2,1.6] |  | 1.1 [0.5,2.5] |  | 2.4 [1.3,4.3] |  |
| Others | 1.3 [1.1,1.6] |  | 0.8 [0.4,1.8] |  | 1.2 [0.6,2.7] |  |
| Residence |  | <.001 |  | .480 |  | .088 |
| Urban | Reference |  | Reference |  | Reference |  |
| Rural | 0.8 [0.7,0.9] |  | 0.8 [0.5,1.4] |  | 0.7 [0.5,1.0] |  |
| Medicaid*WIC registration |  | <.001 |  | .010 |  | .656 |
| Medicaid | 0.6 [0.5,0.7] |  | 0.4 [0.3,0.8] |  | 0.7 [0.4,1.3] |  |
| WIC registration | 0.9 [0.8,1.0] |  | 1.0 [0.6,1.7] |  | 1.0 [0.6,1.6] |  |
| Abuse*Depression |  | <.001 |  | .168 |  | .009 |
| Abuse | 0.7 [0.5,1.0] |  | 0.9 [0.2,5.0] |  | 1.0 [0.5,2.3] |  |
| Depression | 0.8 [0.5,1.3] |  | 0.9 [0.1,7.5] |  | 1.0 [0.3,3.2] |  |
| Marital status*Pregnancy intention*Prenatal vitamins |  | <.001 |  | .008 |  | .560 |
| Unmarried | 0.7 [0.5,0.8] |  | 0.5 [0.2,1.0] |  | 0.6 [0.3,1.4] |  |
| Unintended pregnancy | 0.8 [0.7,1.0] |  | 2.7 [1.3,5.6] |  | 1.1 [0.6,1.9] |  |
| Irregular vitamins | 0.9 [0.7,1.2] |  | 0.3 [0.1,0.8] |  | 1.2 [0.6,2.6] |  |
| Parity |  |  |  |  |  |  |
| Primiparity | 1.8 [1.6,2.1] | <.001 | 1.6 [1.0,2.4] | <.048 | 2.4 [1.6,3.4] | <.001 |
| Multiparity | Reference |  | Reference |  | Reference |  |
| APNCU Index |  | <.001 |  | .722 |  | .137 |
| Inadequate | 0.6 [0.5,0.7] |  | 0.8 [0.4,1.5] |  | 0.7 [0.5,1.2] |  |
| Intermediate | 0.9 [0.8,1.1] |  | 1.2 [0.6,2.2] |  | 0.6 [0.4,1.1] |  |
| Adequate | Reference |  | Reference |  | Reference |  |
| Adequate plus | 1.0 [0.9,1.1] |  | 1.0 [0.6,1.7] |  | 1.1 [0.7,1.7] |  |

*Table continued on the next page*

**S9 Table (continued).** Logistic regression model examining correlates of quitting conventional cigarettes, e-cigarettes, and dual-use during pregnancy, stratified by survey-year (2016–2019).

| **Characteristic** | **Quits cigarettes** | | **Quits e-cigarettes** | | **Quits dual use** | |
| --- | --- | --- | --- | --- | --- | --- |
|  | **AOR*^a^* [95%CI]** | ***p*-value** | **AOR*^a^* [95%CI]** | ***p*-value** | **AOR*^a^* [95%CI]** | ***p*-value** |
| Cigarettes per day |  | <.001 | NA |  |  | <.001 |
| 1-10 | Reference |  |  |  | Reference |  |
| 11-20 | 0.3 [0.2,0.3] |  |  |  | 0.2 [0.1,0.3] |  |
| >20 (one pack) | 0.2 [0.2,0.3] |  |  |  | 0.2 [0.1,0.4] |  |
| E-cigarettes per week | NA |  |  | <.001 |  | .066 |
| Occasional |  |  | Reference |  | Reference |  |
| Some days |  |  | 0.7 [0.3,1.5] |  | 0.8 [0.5,1.4] |  |
| Everyday |  |  | 0.3 [0.2,0.4] |  | 1.4 [1.0,2.0] |  |
| Alcohol |  |  |  |  |  |  |
| None to occasional | Reference | <.001 | Reference | .863 | Reference | <.001 |
| Moderate | 1.5 [1.2,1.8] |  | 0.9 [0.5,1.5] |  | 2.7 [1.5,4.8] |  |
| Heavy | 1.0 [0.8,1.3] |  | 1.0 [0.4,2.3] |  | 3.2 [1.7,5.9] |  |

*^a^* Reference groups for AOR calculation under each category: age: 20-34, education: high school, some college, no degree, race & ethnicity: White non-Hispanic, residence: urban, Medicaid & WIC registration: none, partner/ ex-partner abuse and depression: none, Obstetric: multiparity, married status, intended pregnancy, regular prenatal vitamins, APNCU index: adequate, cigarettes per day: half a pack or less, e-cigarettes per week: occasional, alcohol: none to occasional.

Abbreviations: AOR – adjusted odds ratio, APNCU ‒ Adequacy of Prenatal Care Utilization, CI – confidence interval, HCW ‒ Health Care Worker, PNC – Prenatal Care, WIC ‒ Women, Infants, and Children.
